# The mRNA level of the transforming growth factor β1 gene, but not the amount of the gene product, can be considered as a potential prognostic parameter in inflammatory bowel diseases in children

**DOI:** 10.1007/s00384-012-1489-4

**Published:** 2012-05-15

**Authors:** Anna Liberek, Zbigniew Kmieć, Dorota Kartanowicz, Piotr M. Wierzbicki, Marcin Stanisławowski, Lucyna Kaszubowska, Grażyna Łuczak, Magdalena Góra-Gębka, Piotr Landowski, Agnieszka Szlagatys-Sidorkiewicz, Tomasz Liberek, Barbara Kamińska, Joanna Jakóbkiewicz-Banecka, Grzegorz Węgrzyn

**Affiliations:** 1 Department of Pediatrics, Pediatric Gastroenterology, Hepatology and Nutrition, Medical University of Gdańsk, Nowe Ogrody 1-6, 80-803 Gdańsk, Poland; 2 Department of Histology, Medical University of Gdańsk, Debinki 1, 80-211 Gdańsk, Poland; 3 Department of Nephrology, Transplantology and Internal Medicine, Medical University of Gdańsk, Dębinki 7, 80-211 Gdańsk, Poland; 4 Department of Molecular Biology, University of Gdańsk, Kładki 24, 80-822 Gdańsk, Poland

**Keywords:** Inflammatory bowel disease, Transforming growth factor β1 (TGF-β1), Children, Gene expression

## Abstract

**Purpose:**

Transforming growth factor β1 (TGF-β1) plays a role in cell proliferation and differentiation, and it can modulate immune response. In this work, we asked whether levels of either TGF-β1 or mRNA of the corresponding gene in plasma or tissue can be useful in diagnosing and/or monitoring of the clinical course of inflammatory bowel diseases (IBD).

**Methods:**

The study group consisted of 104 pediatric patients with IBD: 36 with Crohn’s disease (CD) and 68 with ulcerative colitis (UC); 42 children represented the control group. TGF-β1 levels in plasma and intestinal mucosa were estimated by ELISA and immunohistochemistry (IHC), respectively. Levels of *TGF-β1* mRNA were determined by reverse transcription and real-time PCR.

**Results:**

In patients with IBD, and in subgroups with CD and UC, no significant differences in the TGF-β1 level in plasma and tissue were found relative to the control group. These variables were not dependent on the stage of the disease, its activity or severity of endoscopic and histopathological findings. *TGF-β1* mRNA levels were significantly higher in tissue samples withdrawn during the relapse of the disease than in those taken during the remission or in the control group. However, no correlation between TGF-β1 plasma levels and *TGF-β1* mRNA amount in the intestinal mucosa was observed.

**Conclusions:**

The *TGF-β1* mRNA level, but not the amount of the gene product, was significantly increased in the pathologically changed tissue during the relapse of IBD. We suggest that this parameter might be considered as a potential prognostic value when assessing IBD in children.

**Electronic supplementary material:**

The online version of this article (doi:10.1007/s00384-012-1489-4) contains supplementary material, which is available to authorized users.

## Introduction

Transforming growth factor β (TGF-β), especially its isoform 1, is known to be one of the major factors involved in immunological homeostasis. Biological activity of TGF-β consists of effects on cell proliferation and maturation or immunomodulation [1–3]. Within particular organs and tissues, TGF-β is involved in regenerative processes and upon some conditions may be responsible for pathological fibrosis [4–6]. Long-term immunoregulatory disturbances are proved to play a crucial role in the pathogenesis of numerous diseases [4–9], thus TGF-β appears to be of great interest in contemporary studies. Either insufficiency or hyperactivity of TGF-β may be responsible for some diseases, particularly due to its influence on immune response and regenerative processes. Some clinical and experimental studies indicated the role of TGF-β in chronic inflammatory diseases, especially those of the bowels [10–14].

In the course of chronic inflammatory bowel diseases, the cicatricial changes within the bowel appear to be related to the activity of TGF-β, which is responsible for the fibrogenesis. However it still remains unknown whether an increased amount of TGF-β in the inflamed tissue is the cause or only an innocent response for the insult and damage of the tissue related to the disease. Damage of the tissue is known to be one of the major factors responsible for the activation of the TGF-β1 gene and increased production of this cytokine in site [15, 16]. One may also assume that chronic inflammation is determined by the deficiency of TGF-β biological activity. It was reported that mice lacking TGF-β activity presented with multi-organ inflammatory and necrotic changes shortly after birth [12, 17].

Within the immune system, TGF-β1 plays an immuno-suppressive role preventing autoimmune and chronic inflammatory diseases. It inhibits the processes of activation of auto-reactive lymphocytes T, production of auto-antibodies, and thus, the self-antigens damage [18, 19]. The wide range of data indicates the main role of altered function of cytokines, including TGF-β1, in the pathogenesis of chronic inflammatory diseases, thus, further studies, especially in children, appear to be warranted [20, 21]. The aim of the study was to evaluate the role of TGF-β1 in the pathogenesis of inflammatory bowel diseases (IBD) and to assess whether levels of either TGF-β1 and/or mRNA of its gene, in either plasma or tissue expression, can be useful in diagnosing and/or monitoring of the clinical course of these diseases.

## Methods

### Patients

The study group consisted of 104 patients with IBD, including 36 with Crohn’s disease (CD) and 68 with ulcerative colitis (UC), at the age 1.5–18.4 years (mean, 13.0 ± 4.5; median, 14.5). Forty-two children represented the control group, which consisted of 20 girls and 22 boys at the age 2.0–18.0 years (mean, 11.0 ± 5.0; median, 11.0), who underwent endoscopy because of the events of the gastrointestinal (GI) bleedings (this relatively high number of pediatric patients with GI bleedings resulted from the fact that the Department of Pediatrics, Pediatric Gastroenterology, Hepatology and Nutrition of the Medical University of Gdańsk serves as one of pediatric gastroenterology reference centers for Northern Poland, covering relatively large population); in this group of patients, inflammatory processes, immune disorders, malignancies, and nutritional abnormalities were excluded. None of the patients received immuno-modulating drugs at least 6 months prior to the study. Most IBD patients were treated with anti-inflammatory drugs (e.g., 5-amino salicylic acid). Only minority, with more severe disease course or during the relapse, used immunosuppresants (e.g., steroids or azathioprine). However, since we have detailed data on drug therapies only for a part of patients, this parameter was not analyzed in this study.

### Clinical parameters and samples’ collection

Endoscopic and histological classifications of intestinal mucosa were performed according to the Porto criteria [22]. Among IBD patients, severities of CD and UC were estimated according to the Hyams scale (Pediatric Crohn’s Disease Activity Index) and the Truelove–Witts scale, respectively [23, 24].

In patients in the acute phase of the IBD, both blood and bowel tissue samples were obtained while in those in remission, the level of TGF-β in the serum was assessed. In a few patients, when the control endoscopy was mandatory, the level of TGF-β was measured also in the bowel tissue. All samples were obtained while performing obligatory diagnostic procedures.

### Inflammatory parameters

The following inflammatory parameters were determined for all subjects: C-reactive protein level (CRP), erythrocytes sedimentation rate, and full blood count.

### Determination of TGF-β1 level

The TGF-β1 level in plasma was estimated by ELISA (Quantikine TGF-β1 R&D Systems, USA), according to manufacturer’s instructions. Amount of the TGF-β1 protein in the intestinal mucosa was estimated by immunohistochemistry (IHC; Vectastain ABC Kit, Vector Laboratories, USA) and microscopic observations at the final magnifications of ×200 and ×400. The results were described in a semi-quantitative scale (0, 1+, 2+, and 3+).

### Measurement of mRNA levels

The mRNA level in the intestinal tissue was determined by reverse transcription and Real-Time PCR. Total RNA was isolated by employing the Total RNA Prep Plus kit (A&A Biotechnology, Poland), according to manufacturer’s instructions. Concentration and quality of RNA samples were determined spectrophotometrically (Smart Spec 3000 apparatus, Bio-Rad, USA). One microgram of RNA, 0.25 μg of oligo T_18_, and M-MLV enzyme (Promega, Madison, WI, USA) were used for reverse transcription in total volume of 10 μl. The qPCR reaction contained 0.4 μl cDNA, 180 nM each primer and iQSybrGreen Supermix kit (Bio-Rad) and was performed in iCycler iQ (Bio-Rad) in total volume of 20 μl. Primers for *TGF-β1* and *ACTB* were designed de novo using Primer3Plus based on BLAST, ENSMBL, and AceView databases; TGF-β1: 5′-CAG CAA CAA TTC CTG GCG ATA CC and TGFβ-2, 5′-CGA AAG CCC TCA ATT TCC CCT C, bact1-1, 5′-TGT GCC CAT CTA CGA GGG GTA TGC, and bact1-2, 5′-GGT ACA TGG TGG TGC CGC CAG ACA. The reactions were run in triplicates and the obtained data were averaged followed by data analysis calculated with iQ ver. 3.1 software (Bio-Rad). The *ACTB* gene was used as a control to normalize the values in common ΔΔ*C*_t_ quantification method [25, 26].

### Statistical analysis

Statistical analysis of the results was performed using non-parametric tests: the Mann–Whitney test for comparison of two groups, the ANOVA Kruskal–Wallis test for comparison of several groups, and the Spearman correlation test. For qualitative parameters, the Pearson’s *χ* ^2^ test was used, with Yates correction if *n* < 10. The results were considered statistically significant when *p* < 0.05. The calculations were performed employing Statistica 7 software (StatSoft Inc., OK, USA).

### Ethical considerations

This study was approved by the Independent Bio-Ethical Committee for Research at the Medical University of Gdańsk (NKEBN/13/2004). Informed consent forms of patients and controls (or their parents) were obtained.

## Results

### TGF-β1 plasma level

In the studied group of patients, the TGF-β1 plasma levels were in the range of 0.11–55.10 ng/ml (mean, 7.01 ± 9.37 ng/ml; median, 3.51 ng/ml) (Table 1). No statistically significant differences between the TGF-β1 plasma levels in IBD patients and control group were noted, although *p* value was 0.06. No statistically significant differences were also observed between the UC and the CD patients and the control group (*p* = 0.16) (Table 1).

**Table 1:** Plasma concentration of TGF-β in the study groups and subgroups

Group	Number	TGF-β1 concentration (ng/ml)^a^				
		Mean	Median	Min	Max	SD
Control	42	6.96	2.79	0.54	55.10	11.61
IBD	104	7.71	3.72	0.20	43.14	9.05
UC	68	7.86	3.54	0.20	43.14	9.83
CD	36	7.46	4.43	0.72	30.45	7.49

Statistical analysis: control vs. IBD (*p* = 0.06); CD vs. UC (*p* = 0.16)

^a^Results from three independent measurements in each sample (including RNA extraction) are presented

*SD* standard deviation

No statistically significant differences were noted among the TGF-β1 plasma levels in the particular phases of the disease (mean TGF-β1 plasma levels were as follows: at the time of diagnosis, 8.59 ng/ml; at the relapse, 7.22 ng/ml; and at the remission, 7.91 ng/ml), and in the control group (6.3 ng/ml; *p* = 0.22), as well as in the particular sub-groups, the UC (*p* = 0.55) and the CD (*p* = 0.65) patients. Moreover, no statistically significant differences were found between TGF-β1 plasma levels at particular activity grades of IBD relative to the control group (*p* = 0.27). The same was true when UC and CD were considered separately, relative to the control (*p* = 0.13 and 0.23, respectively).

In the studied group of children with IBD, the TGF-β1 plasma levels showed no correlation with endoscopic (*p* = 0.42) and histopathological (*p* = 0.55) activity of the disease. Furthermore, no statistically significant correlations of TGF-β1 plasma levels with CRP, SR, WBC, and platelet count were noted (data not shown).

### TGF-β1 levels in the intestinal tissue

The IHC studies were performed in intestinal tissue samples in 72 children (IBD, 53; UC, 37; CD, 16; and the control group, 19). The intensities of the IHC reaction specific to TGF-β1 were assessed in the semi-quantitative scale as follows: 1+ in 15 (19.2 %) children, 2+ in 39 (50 %), and 3+ in 24 patients (30.8 %). No statistically significant differences were noted between the intensity of IHC in the IBD patients and in the control group and in the particular groups of CD and UC patients (data not shown).

In the studied group of children, the intensity of IHC showed no correlation with the disease activity neither in the whole IBD group nor in the particular groups of CD and UC patients (data not shown).

### mRNA levels of the *TGF-β1* gene

The mRNA levels of the *TGF-β1* gene in the intestinal tissue in the studied group of children are presented in Table 2. The levels in the inflamed intestinal tissue were significantly higher in IBD patients than in the control group (*p* = 0.002). However, the mRNA levels in the tissue samples obtained from macroscopically unchanged intestinal regions in the IBD patients in the active phase of the disease showed no significant differences relative to the control group (*p* = 0.12).

**Table 2:** *TGF-β1* mRNA levels in intestinal tissue of the control group and the study groups

Sample	Number	*TGF-β1* mRNA level (arbitrary units)^a^				
		Mean	Median	Min.	Max	SD
Control	42	0.08596	0.04028	0.00103	0.82469	0.16012
IBD-affected area	90	0.15040	0.06569	0.00068	1.59320	0.21443
UC	57	0.15512	0.07289	0.00068	0.85378	0.18372
CD	33	0.15782	0.06569	0.00296	1.59320	0.27999
IBD-unaffected area (active stage of disease)	41	0.03852	0.02244	0.00108	0.30119	0.06087

^a^Results from three independent measurements in each sample (including RNA extraction) are presented. Statistical analysis: control vs. IBD (affected area; *p* = 0.002); control vs. IBD (unaffected area; *p* = 0.12); control vs. UC (affected area; *p* = 0.02); control vs. CD (affected area; *p* = 0.05); UC vs. CD (affected area; *p* = 1.0)

Like in the whole IBD group, in the UC patients the mRNA levels in the macroscopically changed intestinal tissue were statistically higher than in the control group (*p* = 0.02), and at the edge of statistical significance in the CD patients in comparison with the control group (*p* = 0.05). No statistically significant differences were noted between the mRNA levels in the groups of CD and UC patients (*p* = 1.00). Furthermore, the mRNA levels of the *TGF-β1* gene in tissue samples obtained from patients in the active phase of the disease, but from the macroscopically unchanged intestinal regions, showed no statistically significant differences with the control group and in both CD and UC groups (*p* = 0.26).

**Fig. 1:**
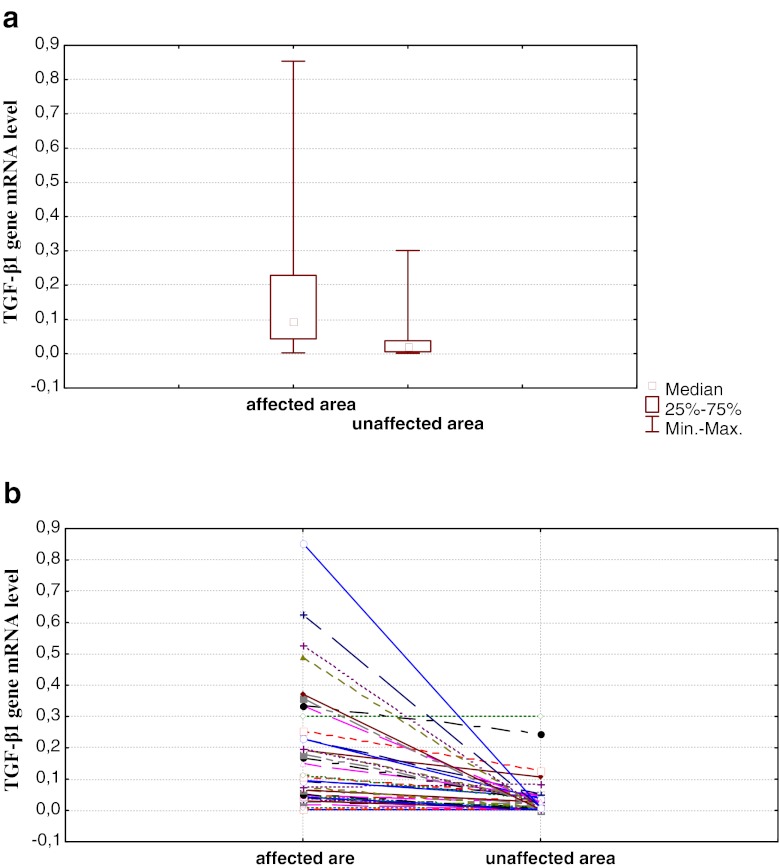
*TGF-β1* mRNA level in samples from the affected and unaffected areas during active stage of disease in the IBD group (collective data). **a** Values for the whole group and **b** results for individual patients. Results from three independent measurements in each sample (including RNA extraction) are presented. In (**a**), the difference between values presented for affected and unaffected areas was statistically significant (*p* < 0.001)

Moreover, we have analyzed samples from 41 IBD patients (28 UC patients and 13 CD patients) in which samples of the intestinal tissue were withdrawn during colonoscopy at the active phase of the disease from both changed and unchanged areas (Fig. 1). Statistically significant differences were found between *TGF-β1* mRNA levels in changed and unchanged tissues when the whole group of IBD was analyzed (*p* < 0.001) as well as in the CD and UC subgroups (*p* < 0.001 in both cases), namely, the levels in the changed tissue samples were higher than in unchanged tissue samples (Fig. 1).

**Fig. 2:**
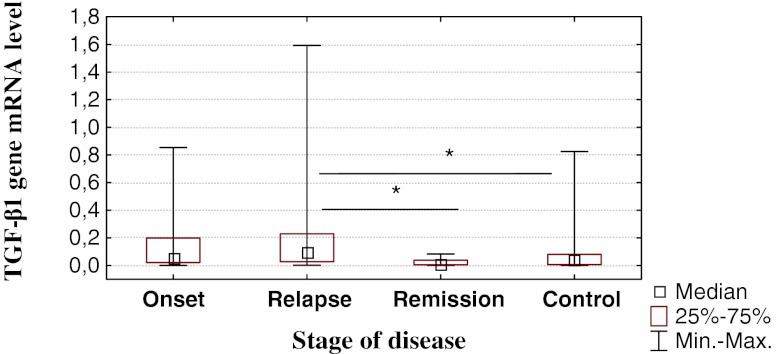
*TGF-β1* mRNA level at different stages of the disease in IBD patients and in the control group. Results from three independent measurements in each sample (including RNA extraction) are presented. Statistically significant differences are marked by *asterisks* (the *p* values were 0.01 and 0.04 for comparison between relapse vs. remission and remission vs. control, respectively)

The comparison of *TGF-β1* mRNA levels in particular phases of the disease (Fig. 2) showed significantly higher values in the relapse of the disease relative to both remission (*p* = 0.01) and the control group (*p* = 0.04). However, no statistically significant differences were noted between the mRNA levels in the remission and the control group (*p* = 0.99), and between the mRNA levels in samples withdrawn at the time of diagnosis and (1) the time of remission (*p* = 0.99), (2) the relapse (*p* = 0.12), and (3) the control group (*p* = 0.80).

No statistically significant differences were noted in the whole IBD group, and in both CD and UC subgroups, when the mRNA levels in macroscopically changed intestinal tissues at the active phase of the disease were tested in relation to the endoscopic (IBD, *p* = 0.42; CD, *p* = 0.40; and UC, *p* = 0.32) and histopathological (IBD, *p* = 0.55; CD, *p* = 0.95; and UC, *p* = 0.23) changes.

Interestingly, no statistically significant correlation was observed between the TGF-β1 plasma levels and the *TGF-β1* mRNA levels in the macroscopically changed intestinal tissue in the group of IBD patients (*R*_s_ = 0.19; *p* = 0.07) and in both CD and UC subgroups (for CD, *R*_s_ = 0.07; *p* = 0.71 and for UC, *R*_s_ = 0.24; *p* = 0.08).

## Discussion

The wide range of cytokines plays a significant role in the pathogenesis of the chronic inflammatory bowel diseases, thus, intensive research estimating their levels either in the blood or in the inflamed tissues employing immunohistochemical and molecular methods, is being conducted [20, 27–31]. Furthermore, signaling pathways of particular cytokines are being assessed at the molecular level [32, 33]. Most of the data are related to the blood levels of particular cytokines due to the less invasive procedures of obtaining blood samples comparing to obtaining tissue samples and availability of numerous ELISA kits suitable for estimation of cytokine levels in the blood [20, 28, 31, 33].

Data referring the role of TGF-β1 in the pathogenesis of the chronic inflammatory bowel disease, especially in children, are rather scarce [20, 21]. In the group of children described in this report and suffering from chronic inflammatory bowel diseases, the TGF-β1 plasma levels showed no significant differences in both CD and UC patients and the control group. Medical reports on this topic are equivocal. Sturm et al. [34] presented no statistically significant differences between the TGF-β1 and HGF (hepatocyte growth factor) plasma levels in patients with IBD and the control group. However, in other study, similarly to our results, the tendency to the higher TGF-β1 plasma levels in the IBD patients was noted [34]. No statistically significant differences between the TGF-β1 plasma levels in patients with CD and children with UC, were also noted in the research by Kader et al. [20]. In that study, plasma levels of over 70 cytokines and growth factors were estimated in the group of 88 children with IBD (65 with UC and 23 with CD). In patients with CD, plasma levels of four cytokines, PLGF (placenta growth factor), IL-7, Il-12p40, and TGF-β1 were significantly higher in the remission than in the active phase of the disease. However, in patients with UC, such differences were proved to be related only to one cytokine, IL-12p40 [20]. Those authors put stress on the distinct profile of the cytokines’ activity in patients with CD and patients with UC, which may be mainly explained by Th1 cell involvement in the immune response in CD patients, and Th2 in UC patients. Furthermore, no decrease in the cytokines’ plasma levels in the course of the clinical improvement was noted [20].

In the group of patients studied in this work, the TGF-β1 plasma levels showed no correlation with the clinical phase of the disease. It might be partly explained by rather low severity of the disease at the time of diagnosis in the studied group of children. Sturm et al. [34] presented no significant differences between TGF-β1 plasma levels in 74 patients with IBD (28 with UC, 45 with CD) in relation to particular phases of the disease and to the control group. Our data showed also no significant correlation between the TGF-β1 plasma levels and the results of the laboratory blood tests.

Besides the TGF-β1 plasma levels, the intestinal tissue levels of TGF-β1 were estimated by IHC. No statistically significant differences were noted between the intensity of IHC in the IBD patients and in the control group, and in particular subgroups of CD and UC patients. Previously, Lawrence et al. [35] observed the diverse pattern of the IHC intensity in relation to the type of IBD: in the patients with CD, the intramural IHC was the most intense, while in the patients with UC the highest signals were detected in submucosal and within the lamina propria. The intensity of the IHC correlated with the inflammatory infiltration in all patients and it finally appeared to be the main factor affecting the IHC [35]. Numerous authors suggested a role for some other TGF-β isoforms in the pathogenesis of IBD. Kazanawa et al. [36] showed no intestinal tissue expression of the *TGF-β1* gene in adults with CD and UC. However in some of them, TGF-β2 and TGF-β3 isoforms were detected along with the intestinal tissue production of FGF (fibroblast growth factor) and VEGF (vascular endothelial growth factor) [36]. Noteworthy, McKaig et al. [37] reported a similar expression of the *TGF-β1* gene in cell cultures of myofibroblasts obtained from macroscopically unchanged or inflamed intestinal regions, in both CD and UC patients. Furthermore, the presence of other TGF isoforms was detected in cell cultures of myofibroblasts obtained from inflamed intestinal regions: TGF- β2 mainly in CD patients and TGF-β3 in UC patients [37]. In intestinal myofibroblast cultures from CD patients, intense cell proliferation, along with increased tissue inhibitors of metalloproteinases production was observed, what appeared to be responsible for the predisposition to fibrosis and intestinal strictures in this group of patients [38].

Our data showed that the mRNA levels in the intestinal tissue were significantly higher in the IBD patients in the active phase of the disease than in the control group. These differences were prominent when tissue samples were obtained from the macroscopically changed regions. The tissue samples obtained from the patients in the active phase of the disease, but from the macroscopically unchanged intestinal regions, showed no statistically significant differences relative to the control group. This was also true for both CD and UC subgroups. Del Zotto et al. [39] showed similar relationships estimating the TGF-β1 levels in cell cultures. Contrary to the CD patients, monocytes and lymphocytes T, obtained from the lamina propria of UC patients, produced more TGF-β1 than control cells [39].

It is intriguing that despite the above mentioned differences in the levels of *TGF-β1* mRNA, no such differences were detected in levels of the gene product (the TGF-β1 protein) in both plasma and tissue samples. The lack of correlation between the tissue mRNA and the plasma protein levels might be explained by possible specificity of the effects for *TGF-β1* expression, restricted solely to the pathologically changed tissue. Such a hypothesis may be corroborated by the fact that *TGF-β1* mRNA levels in the inflamed intestinal tissue were significantly higher in IBD patients than in the control group, while analogous values measured in tissue samples obtained from macroscopically unchanged intestinal regions of the same patients showed no significant differences relative to the control group. A lack of the difference in the levels of the TGF-β1 protein between IBD patients and the control group, in the light of significantly different corresponding mRNA levels, could arise from the use of a semi-quantitative method for determination of the protein level. Alternatively, such a discrepancy might result from a putative, extensive post-transcriptional regulation of the *TGF-β1* gene expression.

Our results indicated that *TGF-β1* mRNA levels in the intestinal tissue were significantly higher in tissue samples obtained at the time of relapse than in tissue samples obtained at the time of diagnosis. This may be due to the signaling pathways disturbances and defects of regulatory mechanisms essential in the active phase of the disease [40]. Since serial probing in different activity stages of the disease is not recommended if there is no therapeutic consequences, our results cannot be applied directly in estimating the progress of the disease. However, one can speculate that if tissue samples were withdrawn during medical procedures anyway, then estimation of *TGF-β1* mRNA levels might be considered as a potential prognostic value of the course of the disease, indicating unfavorable prognosis in the case of increased levels.

## Electronic supplementary material

Below is the link to the electronic supplementary material.

ESM 1 (PDF 1904 kb)

ESM 2 (PDF 2002 kb)
