# Supplementary material for: The mRNA level of the transforming growth factor β1 gene, but not the amount of the gene product, can be considered as a potential prognostic parameter in inflammatory bowel diseases in children
Source: Int J Colorectal Dis. 2012 May 15;28(2):165–72. doi: 10.1007/s00384-012-1489-4 (PMC3579420; doi:10.1007/s00384-012-1489-4)
Supplement: Supplementary file 1 — (PDF 1904 kb) [file 384_2012_1489_MOESM1_ESM.pdf]

Katedra i Klinika Pediatrii, Gastroenterologii, Hepatologii i Żywienia Dzieci GUM
Pomorskie Centrum Traumatologii Wojewódzki Szpital Specjalistyczny w Gdańsku
ul. Nowe Ogrody 1-6
Pracownia Endoskopii

Informacja dla Pacjentów i Rodziców
(Opiekunów ustawowych)

Imię i nazwisko pacjenta:...

Numer badania:...

Data:..

Oddział...

Kolonoskopia

Szanowna Pani! Szanowny Panie!

Zaproponowane Państwa dziecku badanie endoskopowe (ocena jelita grubego) ma na celu wyjaśnienie przyczyny dolegliwości dziecka i tym samym zwiększy szansę na skuteczną terapię.

Chcielibyśmy abyście Państwo zostali poinformowani o tym jak należy przygotować się do badania, jaki jest jego przebieg oraz o niewielkim związanym z tym badaniem ryzyku powikłań i następnie dobrowolnie wyrazili zgodę na badanie u dziecka.

Przygotowanie do badania

Polega ono na oczyszczeniu jelita grubego. Jeżeli badanie planowane jest na godziny poranne całe przygotowanie powinno odbyć się w godzinach popołudniowych i wieczornych dnia poprzedniego. Od dnia poprzedzającego należy powstrzymywać się również od obfitych posiłków.

Dopuszczalne jest spożycie na śniadanie i obiad lekkostrawnych, bezresztkowych posiłków, tzn. składających się z półpłynnych, całkowicie wchłanialnych produktów (np. kisiel, budyń, kaszka, jogurty, zupa klarowna). W godzinach obiadu zwykle przyjmowany jest już preparat przeczyszczający. Przez cały dzień możne dziecko pić do woli różne

płyny (herbata, woda, soki). Dziecko powinno przyjmować zalecane wcześniej leki. Modyfikacji postępowania mogą wymagać chorzy na cukrzycę, zwłaszcza przyjmujący insulinę. Każdorazowo sposób postępowania należy indywidualnie uzgodnić z lekarzem. W dniu badania nie wolno już jeść ani pić. Pusty żołądek wskazany jest na wypadek podania dożylnych środków znieczulających.

Opis badania

✓ badanie polega na delikatnym wprowadzeniu giętkiego i gładkiego aparatu przez kanał odbytu do poszczególnych coraz głębszych partii jelita grubego. Licząc od początku jelita grubego, w prawym podbrzuszu, tam gdzie łączy się ono z jelitem cienkim, wyróżniamy kolejno następujące **części jelita grubego**: kątnicę, wstępnicę, następnie po zagięciu się jelita pod wątrobą poprzecznicę i dalej po zagięciu w okolicach śledziony zstępnicę, esicę i odbytnicę. Możliwe jest prześledzić wszystkie te części (w kolejności od końca jelita), ocenić wygląd błony śluzowej, ukształtowanie fałdów i wykluczyć obecność **zmian chorobowych** takich jak: zapalenie błony śluzowej, nadżerki, owrzodzenia, krwawienie, polipy, guzy nowotworowe czy malformacje naczyniowe. Samo wprowadzanie aparatu nie jest bolesne. **Ból** może być wywołany przez pociąganie krezki (silnie unerwiona błona obejmująca jelita) przede wszystkim esicy, w trakcie manewrowania aparatem. Podajemy wówczas środki znieczulające. Nasilenie bólu jest indywidualnie zmienne i zależy od takich cech jak: układ anatomiczny jelita (tzn. mniej lub bardziej skręcona esica), obecność zrostów po przebytych operacjach np. ginekologicznych, osobniczo zróżnicowana wrażliwość na ból. Nieprzyjemnie może też być odczuwane wdmuchiwanie **powietrza**, które umożliwia lekkie rozdęcie jelita i tym samym dokładną ocenę śluzówki. Pod koniec badania zwykle usuwamy nadmiar powietrza. Uczucie dyskomfortu jednak często pozostaje jeszcze na kilka godzin. W razie potrzeby możliwe jest też pobranie za pomocą sondy biopsyjnej, wprowadzanej przez odpowiedni kanał w aparacie, malutkich **wycinków** błony śluzowej do badania histo-patologicznego (mikroskopowego). Umożliwia to precyzyjną ocenę stwierdzonych zmian. Spotkane z kolei **polipy** usuwane są w całości specjalną techniką elektroresekcji, z użyciem pętli diatermicznej. Wszystkie te zabiegi nie są bolesne (błona śluzowa nie ma czucia), wiążą się jedynie z

minimalnym ryzykiem krwawienia. Tylko nieznacznie wydłużają **czas badania**, który zwykle wynosi 20-30 minut.

✓ **Badanie w znieczuleniu ogólnym:** Na badanie w znieczuleniu ogólnym dziecko jest przyjmowane na dwie doby do szpitala. Jeśli badanie przeprowadzane jest w znieczuleniu ogólnym anstezjolog przeprowadzi dodatkowe badanie oraz uzyska dodatkową zgodę. Anestezjolog poda dziecku dożylnie środki zwiotczające i przeciwbólowe. W przypadkach tych zakłada się wkłucie do żyły (zwykle na prawej kończynie górnej – tzw. Venflon), przez który możliwe jest sprawne wykonywanie iniekcji. Dziecko zostanie również zaintubowane (włożenie do tchawicy rurki ułatwiającej oddychanie), co zapobiega również ewentualnemu zachłystnięciu. U dziecka znieczulonego całe badanie odbywa się w pozycji leżącej na plecach. Dziecko nie pamięta całości badania jak i okresu poprzedzającego badania. W trakcie badania dziecko nie odczuwa bólu oraz innych dolegliwości, które mogą powstać w trakcie badania.

Możliwe komplikacje - wysokiej jakości sprzęt, nowoczesna aparatura używana do dezynfekcji endoskopów i sterylizacji akcesoriów oraz duże doświadczenie w wykonywaniu badań jakim obecnie dysponujemy, pozwala na uniknięcie większości z teoretycznie rozważanych i zdarzających się niezwykle rzadko powikłań. Endoskopia jelita grubego należy do badań o niskim ryzyku powikłań. Komplikacje zdarzają się statystycznie raz na kilka tysięcy badań. Należą do nich:

- skaleczenia błony śluzowej i mechaniczne uszkodzenia badanego narządu, perforacje
- zaburzenia ze strony układu sercowo- naczyniowego (zaburzenia rytmu serca, nasilenie bólu okolicy serca)
- reakcje uczuleniowe na użyte środki znieczulające
- przeniesienie zakażenia (w tym wirusowego zapalenia wątroby typu B lub C), co jednak przy nowoczesnych, stosowanych w naszym ośrodku standardach dezynfekcji sprzętu, nie powinno się zdarzyć
- zaburzenia związane ze spadkiem wymiany tlenowej w organizmie.

Informacje o pacjencie

Dla pełnego bezpieczeństwa przeprowadzenia badania prosimy o podanie poniższych informacji:

1. Czy występują u dziecka zaburzenia krzepnięcia krwi (skłonność do krwawień, łatwe powstawanie dużych siniaków, przedłużone krwawienie po skaleczeniu, po ekstrakcji zęba, krwawienie z dziąseł przy myciu zębów

   TAK NIE

2. Czy dziecko przyjmuje leki p/krzepliwe (Sintrom, Acenokumarol, Polopirynę, Aspirynę, Acard, Bestpiryn)

   TAK NIE

3. Czy u dziecka występuje alergia (jeżeli tak to na co ?)

   TAK NIE

4. Czy u dziecka występują choroby serca / układu oddechowego (jeżeli tak to jakie ?)

   TAK NIE

Zgoda Rodzica (Przedstawiciela Ustawowego) i Pacjenta powyżej 16 r.ż.

O przebiegu badania i możliwych komplikacjach zostałem wyczerpująco i zrozumiale poinformowany. Nie mam więcej pytań ani żadnych wątpliwości. Dobrowolnie wyrażam zgodę na przeprowadzenie proponowanego badania.

....

Data Podpis rodzica i dziecka > 16 r.ż. Podpis lekarza

(Przedstawiciela Ustawowego/Opiekuna Faktycznego)
