# Supplementary material for: The mRNA level of the transforming growth factor β1 gene, but not the amount of the gene product, can be considered as a potential prognostic parameter in inflammatory bowel diseases in children
Source: Int J Colorectal Dis. 2012 May 15;28(2):165–72. doi: 10.1007/s00384-012-1489-4 (PMC3579420; doi:10.1007/s00384-012-1489-4)
Supplement: Supplementary file 2 — (PDF 2002 kb) [file 384_2012_1489_MOESM2_ESM.pdf]

AKADEMIA MEDYCZNA W GDAŃSKU
NIEZALEŻNA KOMISJA BIOETYCZNA
DO SPRAW BADAŃ NAUKOWYCH
przy Akademii Medycznej w Gdańsku
80-210 Gdańsk, ul. M. Skłodowskiej-Curie 3 A
telefax 349-10-11

Gdańsk, 2004-03-18

NKEBN/13/2004

Pani
Prof. dr hab. med. Maria Korzon
Kierownik Kliniki Pediatrii, Gastroenterologii
i Onkologii
Instytut Pediatrii
Akademia Medyczna w Gdańsku

W odpowiedzi na zgłoszenie badań z dnia 9.01.2004 r. na temat: **"Rola transformującego czynnika wzrostu ß1 (TGF- ß1) i jego polimorfizmu genowego w etiopatogenezie i przebiegu klinicznym przewlekłych chorób zapalnych przewodu pokarmowego u dzieci"** *(Grant KBN planowany do przeprowadzenia pod kierunkiem dr med. Anny Liberek)* - Niezależna Komisja Bioetyczna do Spraw Badań Naukowych przy Akademii Medycznej w Gdańsku rozpatrzyła ww. zgłoszenie na posiedzeniu w dniu 29 stycznia 2004 r. i – *po uzupełnieniu ww. wniosku zgodnie z zaleceniem Komisji* - wyraża zgodę na prowadzenie badań w zakresie przedstawionym w projekcie pracy stwierdzając, że są to badania poznawcze, dokonywane w interesie pacjentów.

PRZEWODNICZĄCY
Niezależnej Komisji Bioetycznej
do Spraw Badań Naukowych
przy Akademii Medycznej w Gdańsku

prof. dr hab. med. Stefan Raszeja

INFORMACJA O BADANIU ORAZ ŚWIADOMA ZGODA BADANEGO

Program badawczy pt.: „Rola transformującego czynnika wzrostu-β1 (TGF-β1) i jego polimorfizmu genowego w etiopatogenezie i przebiegu klinicznym przewlekłych chorób zapalnych przewodu pokarmowego u dzieci"

Nazwisko i imię pacjenta ....

Data urodzenia..Wiek..............................Płeć.......................

Adres...

Nazwisko i imię rodzica lub prawnego opiekuna dziecka:

....

Adres...

Telefon..

1.Założenia badania.

Badanie ma na celu dokładniejsze poznanie patomechanizmu przewlekłych chorób zapalnych przewodu pokarmowego u dzieci.

Niniejsze badanie zostało przyjęte i zatwierdzone przez Niezależną Komisję Bioetyczną do Spraw badań naukowych przy Akademii Medycznej w Gdańsku, powołaną przez Rektora Uczelni, na podstawie rozporządzenia Ministra Zdrowia i Opieki Społecznej. Zadaniem Komisji jest ochrona praw badanego. Kierownikiem badania jest pani dr med. Anna Liberek, adiunkt w Klinice Pediatrii, Gastroenterologii i Onkologii Dziecięcej AMG.

2. Postępowanie badawcze.

Analizie zostanie poddana krew Państwa dziecka pobrana jednocześnie z próbkami krwi pobieranymi do rutynowych oznaczeń laboratoryjnych (morfologia, CRP, elektrolity, itd.). Ponadto, w trakcie diagnostycznego badania endoskopowego dolnego odcinka przewodu pokarmowego/górnego odcinka przewodu pokarmowego/biopsji wątroby pobrane zostaną bioptaty tkanek celem wykonania analiz objętych niniejszym badaniem, a pobierane rutynowo w ramach diagnostyki schorzenia podstawowego u Państwa dziecka. Badania obejmą też oznaczenie zawartości TGF-β w pokarmie kobiecym w różnych okresach laktacji.

3. Ryzyko związane z badaniem.

Badania wykonywane w ramach realizacji niniejszej pracy nie zwiększają ryzyka związanego z rutynową diagnostyką choroby podstawowej.

4. Korzyści związane z badaniem.

Wyniki przeprowadzonych badań mogą ułatwić w przyszłości diagnozowanie i leczenie przewlekłych chorób zapalnych przewodu pokarmowego. Pomogą też ocenić rolę karmienia naturalnego w zapobieganiu występowania schorzeń zapalnych przewodu pokarmowego u dzieci karmionych mlekiem matki.

dr med. Anna Liberek
specjalista chorób dzieci
1049605

5. Poufność wyników badania.

Zachowamy pełną poufność dotyczącą osobiście Pana/Pani oraz wyników badań. Tajemnicę lekarską zachowamy także w publikacjach naukowych. Jedynie prawomocna decyzja sądu może spowodować ujawnienie danych osobowych. Prawo Państwowe chroni poufność Pana/ Pani danych osobowych.

6. Jak uzyskać dodatkowe informacje.

Zachęcamy Pana/Panią do zadawania pytań, które dotyczą badania, przed i czasie jego przebiegu. Otrzyma Pan/Pani także kopię niniejszego pisma do wglądu. Jeśli Pan/Pani będzie miał dodatkowe pytania prosimy dzwonić na numer 302 25 91 w czasie dnia.

7. Podpisy.

Przeczytałem i zapoznałem się z wyżej podaną informacją oraz możliwością uzyskania dodatkowych informacji, w każdej chwili. Zgadzam się uczestniczyć w badaniu. Otrzymałem jedną kopię niniejszego pisma.

Podpis badanego..Data..................

Podpis rodziców lub opiekuna prawnego. ....Data...............

Ja, niżej podpisany w pełni wyjaśniłem wszelkie szczegóły tego badania choremu lub prawnemu opiekunowi. W pełni świadomie podejmuję się prowadzenia tego badania i rozumiem swoja rolę.

Podpis badacza....................................Nazwisko i imię ....Data.................

Podpis świadka.....................................Nazwisko i imię ....Data...............

Adres i telefon świadka...

dr med. A[illegible] Liberek
specjalista chorób dzieci
10[illegible]05
